# The Wear Behavior of Textured Steel Sliding against Polymers

**DOI:** 10.3390/ma10040330

**Published:** 2017-03-23

**Authors:** Meiling Wang, Changtao Zhang, Xiaolei Wang

**Affiliations:** College of Mechanical and Electrical Engineering, Nanjing University of Aeronautics and Astronautics, Nanjing 210016, China; zhangchangtao0000@163.com

**Keywords:** wear, surface texturing, UHMWPE, POM, PEEK, wear debris

## Abstract

Artificially fabricated surface textures can significantly improve the friction and wear resistance of a tribological contact. Recently, this surface texturing technique has been applied to polymer materials to improve their tribological performance. However, the wear behavior of textured tribo-pairs made of steel and polymer materials has been less thoroughly investigated and is not well understood; thus, it needs further research. The aim of this study is to investigate the wear properties of tribological contacts made of textured stainless steel against polymer surfaces. Three polymer materials were selected in this study, namely, ultrahigh molecular weight polyethylene (UHMWPE), polyoxymethylene (POM) and (polyetheretherketone) PEEK. Wear tests were operated through a ring-on-plane mode. The results revealed that the texture features and material properties affected the wear rates and friction coefficients of the textured tribo-pairs. In general, PEEK/textured steel achieved the lowest wear rate among the three types of tribo-pairs investigated. Energy dispersive x-ray spectroscopy (EDX) analysis revealed that the elements of C and O on the contacting counterfaces varied with texture features and indicated different wear behavior. Experimental and simulated results showed differences in the stress distribution around the dimple edge, which may influence wear performance. Wear debris with different surface morphologies were found for tribo-pairs with varying texture features. This study has increased the understanding of the wear behavior of tribo-pairs between textured stainless steel and polymer materials.

## 1. Introduction

The control of friction and wear are essential to guaranteeing that tribological systems work efficiently, reliably, and durably. Surface texturing, usually involving fabricating well-defined identical features (e.g., dimples) on tribological contacts, is an effective way to reduce friction. The most dominant effect of surface texturing is to provide an additional hydrodynamic lift to increase the load carrying capacity [1], reserve lubricant, and trap wear debris [2]. Surface texturing has been applied in thrust bearings [3,4], journal bearings [5,6], cylinder liners [7], piston rings [8], silicon carbide (SiC) [9,10] and mechanical seals [11,12] to improve the tribological performance of the mating surfaces. However, most studies [13,14,15] focused on the effects surface texturing on friction of stiff counterfaces, while few investigations have been undertaken about its influences on the wear of mechanisms due to surface texturing, especially those of soft counterfaces.

Soft counterface, such as ultrahigh molecular weight polyethylene (UHMWPE), is light and has a good ability of self-lubricating and anti-wear. Counterfaces consisting of stiff and soft materials have been applied in tribological contacts such as artificial joints. Effects of surface texturing on frictional properties of soft counterfaces have attracted great interest for research. Dimples on a polydimethylsiloxane (PDMS) surface can significantly reduce friction [16,17,18]. Micro-dimples on polyoxymethylene (POM) and polypropylene (PP) significantly improved the tribological performance under dry friction [19]. The friction pair consisting of textured 316 stainless steel surfaces against polytetrafluoroethylene (PTFE) presented a reduced friction coefficient [20]. Textured UHMWPE reduced friction and increased wear resistance [21,22]. Different from stiff materials, soft materials usually deform when they are subjected to forces. Thus, the tribological behavior of the counterfaces between stiff and soft materials are speculated to be different from those of stiff materials.

The aims of this study are to investigate the wear behavior of textured stainless steel against polymer materials to understand wear behavior leading to better design of surface textures on soft counterfaces that prolongs the life of tribological contacts. Dimples were fabricated on 304 samples of stainless steel with varying parameters, namely, depth and area density. Wear tests were conducted with ring-on-plane testing. The friction coefficients and wear rates were measured. The worn surface morphologies of the counterfaces and wear debris were examined to assess the wear mechanism. Energy dispersive X-ray spectroscopy (EDX) was performed to investigate changes in the elements of C and O of the contacting counterfaces. Finite element analysis (FEA) was carried out to analyze the stress distribution of the mating contacts.

## 2. Material and Methods

**Figure 1:**
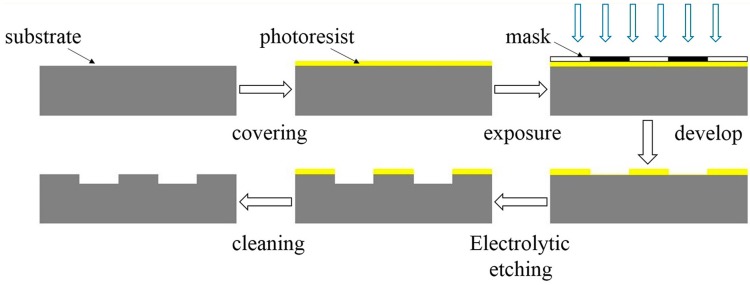
Fabrication process of surface patterns on steel.

**Figure 2:**
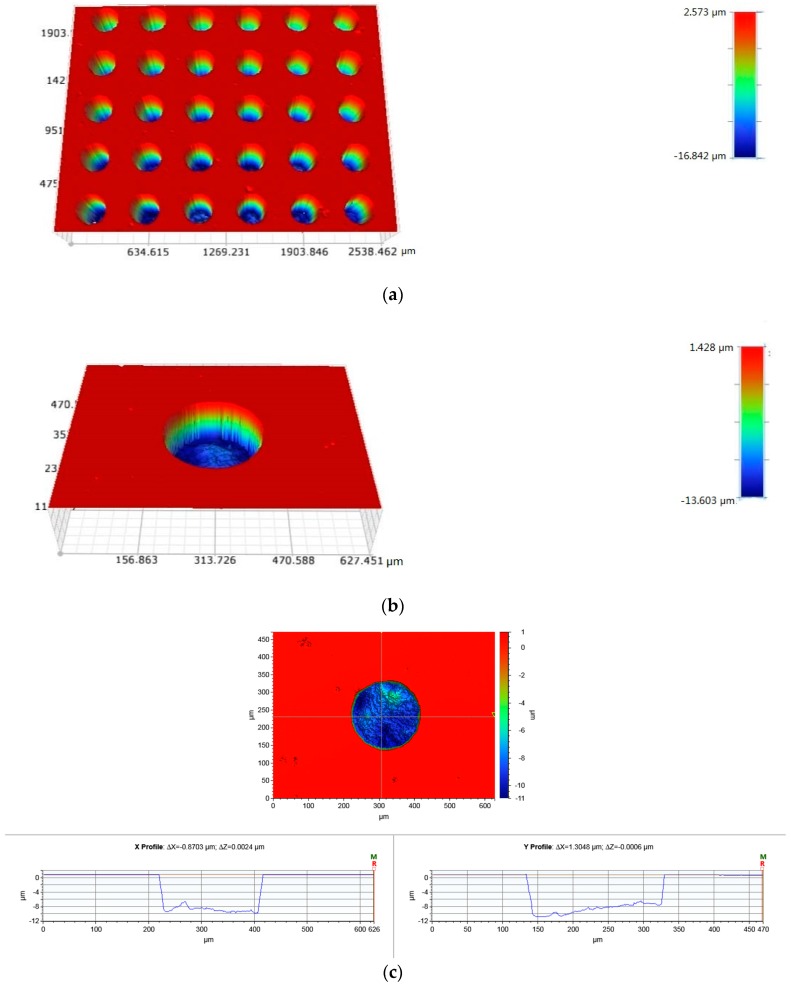
(**a**) The dimples fabricated on the 304 stainless steel; (**b**) a single dimple and (**c**) the cross-section of a dimple.

Lithography-electrolytic technology was used to fabricate surface patterns on the stainless steel. The fabrication process is illustrated in Figure 1. Firstly, the steel was ground and polished (*Sa* ≤ 50 nm). After cleaning the samples, they were evenly coated with a layer of photoresist on the specimens’ surface using spin coating. Then, through exposing and developing, a layer of photoresist mask with certain patterns was formed on the specimen surface. Finally, redundant steel was removed by electrolytic etching to fabricate surface textures. The fabricated dimples are presented in Figure 2. UHMWPE, POM, and (polyetheretherketone) PEEK are widely used polymers because of their low friction and superior mechanical properties and were studied in this work (Table 1). The geometrical parameters of the dimples are shown in Table 2.

**Figure 3:**
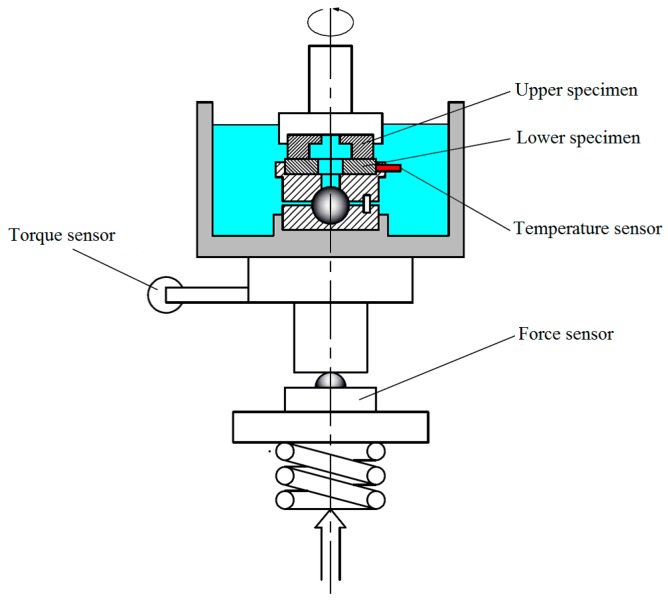
Schematic representation of the test rig.

Friction and wear tests were carried out using a ring-on-disc configuration commercial test rig (Figure 3). The load was set at 70 N at the rotational speed of 0.44 m/s. The reason for choosing a normal force 70 N is that polymer materials are usually soft and undergo plastic deformation under high stress. Therefore, a relatively small force was applied to the tribo-pairs. The Hertzian contact pressure (MPa) was declared, which was 0.15 MPa for non-textured tribo-pairs, 0.167 MPa, 0.188 MPa and 0.25 MPa for textured tribo-pairs with an area density of 10%, 20% and 40%. The lubricant was deionized water. The test duration was 6 hours. The surface morphologies of the mating surfaces after wear were observed using an electronic microscope (Keyence, VHX-500, Osaka, Japan) and a 3D surface profiler (Bruker, contourGT-K, Billerica, MA, USA). EDX was operated to analyze the chemical composition of the tribological contacts. After the wear tests, the lubricants were collected and filtered, and thus the wear debris were collected. The wear debris were imaged using a scanning electron microscope (Sigma 500, Carl Zeiss, Jena, Germany) to study their morphologies. The mean wear rate was determined by weighing the samples before and after each test. FEA was performed with the ANSYS software to simulate the contact situation of the dimple edge. The lower sample was set as a regular hexahedral structure. Sweep meshing method was chosen. The upper sample was set as an irregular rectangle with a cylindrical dimple. The grid cell size was set as 15 μm. The frictional contact type was chosen to simulate the contact behavior of real working conditions. The Coulomb friction coefficient was set as 0.2 in FEA.

## 3. Results

As stated above, this work was performed to understand the wear mechanisms of textured steel sliding against polymer materials. The friction properties and wear rates of non-textured and textured tribo-pairs were obtained and compared. In parallel, the surface morphologies of the counterfaces and wear debris were imaged. EDX revealed the changes in the elements C and O of the tribo-pairs after wear. Finite element simulation provided insights into the contacting conditions of the textured tribo-pairs.

### 3.1. Friction Property

**Figure 4:**
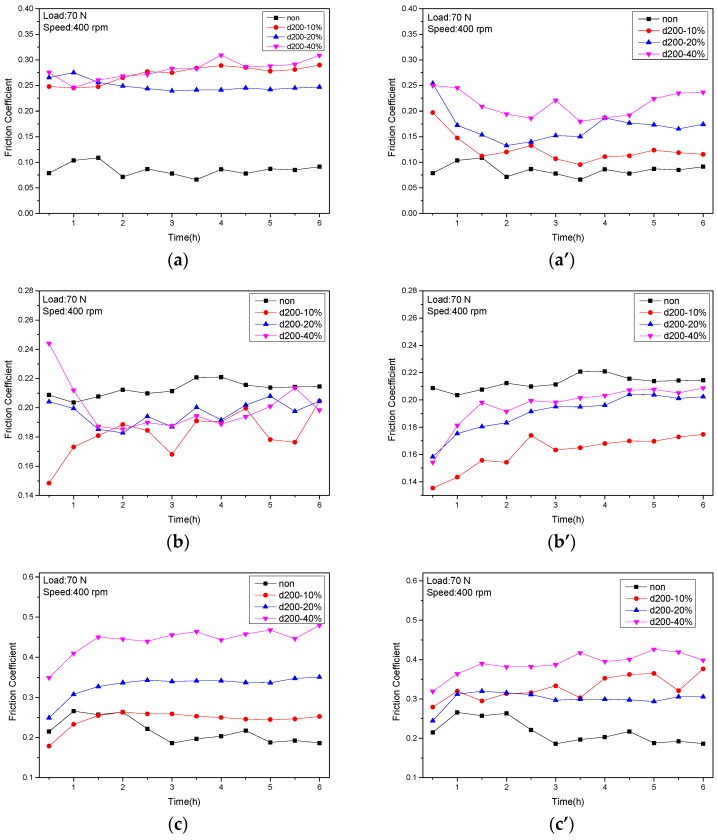
The friction coefficient of textured steel against UHMWPE (**a**,**a’**); POM (**b**,**b’**) and PEEK (**c**,**c’**) at dimple depth of 10 μm (**a**–**c**) and 5 μm (**a’**–**c’**).

Figure 4 displays the friction coefficients of the tribo-pairs made of stainless steel against UHMWPE, POM, and PEEK. Figure 4a,a’ presents the friction coefficients of the tribo-pairs of steel sliding against UHMWPE when the dimple depths were 10 μm and 5 μm, respectively. Compared to the untextured tribo-pair, the friction coefficient of the textured tribo-pair was increased. The tribo-pair with a dimple depth of 5 μm had a smaller friction coefficient value than did that with a dimple depth of 10 μm. Figure 4b,b’ revealed the friction coefficient of POM sliding against steel. POM/textured steel showed friction-reducing effects compared to untextured ones. POM/steel (dimple depth of 5 μm, area density of 10%) had the minimum friction coefficient. Figure 4c,c’ shows the friction coefficients of PEEK/steel. PEEK/textured steel had increased friction compared to non-textured ones. The tribo-pair of PEEK/steel (dimple depth of 10 μm, area density of 40%) had the maximum friction coefficient. Although POM/untextured steel revealed the highest friction among the untextured tribo-pairs, POM/textured steel presented the lowest friction. These results indicate that surface textures play an important role in the friction of the tribo-pairs studied.

### 3.2. Wear Rates

**Figure 5:**
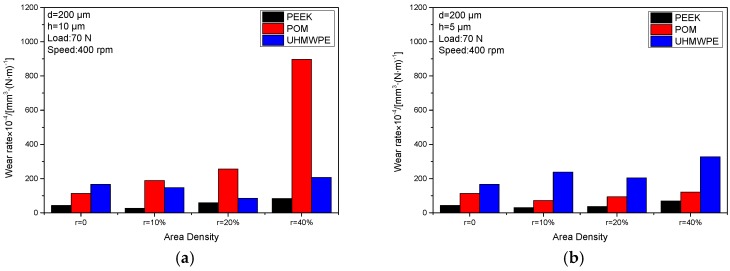
The wear rates of textured steel against PEEK, POM and UHMWPE (*d*: dimple diameter; *h*: dimple depth) at the dimple depth of (**a**) 10 μm and (**b**) 5 μm.

Figure 5 reports the wear rates of steel sliding against PEEK, POM, and UHMWPE. For untextured tribo-pairs, the wear rates decreased with the increment of the elastic modulus of the counterfaces. It can be seen that the tribological pairs of PEEK sliding against stainless steel had the lowest wear rate in the untextured and all textured conditions.

At the dimple depth of 10 μm, the UHMWPE/textured steel with dimples at an area density of 10% and 20% showed reduced wear rates compared to non-textured tribo-pairs, and this increased at a dimple area density of 40%. The POM/textured steel presented increased wear. The PEEK/textured steel with dimples with an area density of 10% decreased and increased with the increment of dimple area density compared to untextured tribo-pairs. Overall, the wear rates of UHMWPE were higher than those of PEEK but lower than those of POM.

At the dimple depth of 5 μm, the POM/textured steel and PEEK/textured steel with dimple area densities of 10% showed reduced wear rates and increased dimples area densities of 20% and 40%, respectively. The UHMWPE/textured steel revealed increased wear at all dimple area densities. The wear rates of POM were higher than those of PEEK but lower than those of UHMWPE. Based on the results, it can be ascertained that the surface parameters, dimple depths, and dimple area densities are crucial factors in influencing the wear performance of the tribo-pairs presented.

### 3.3. Surface Morphologies

**Figure 6:**
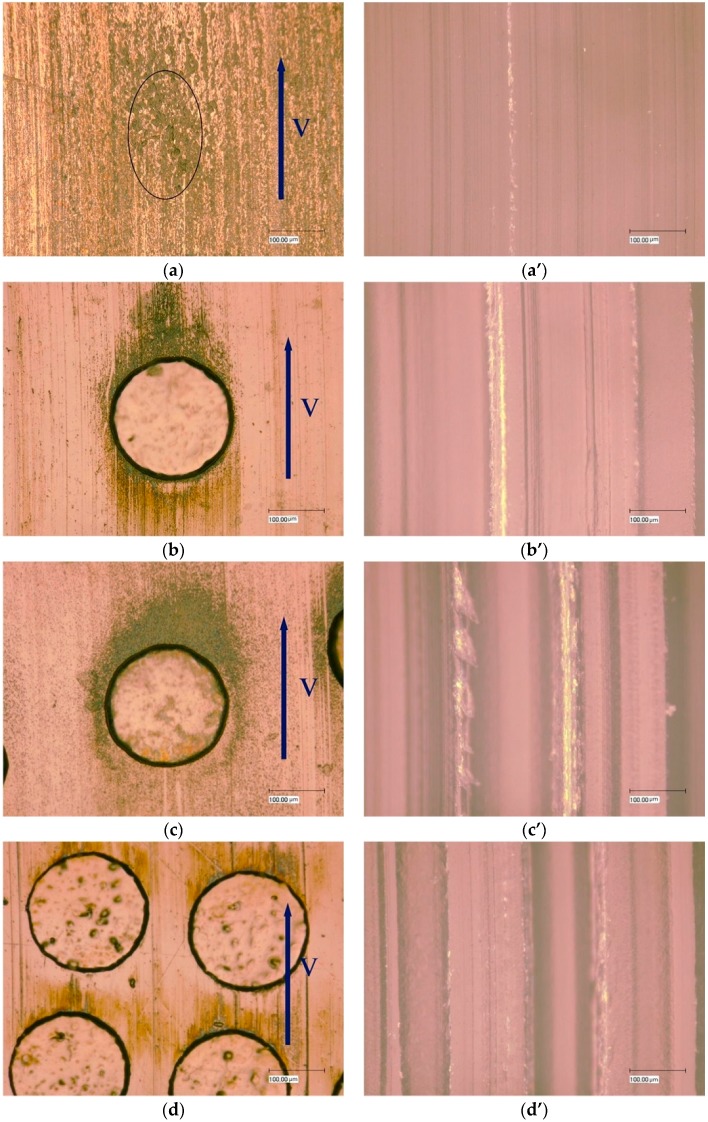
The surface morphologies of 304 stainless steel (**a**–**d**) after wear and the corresponding POM counterface (**a’**–**d’**) when dimple depth was 10 μm: (**a**,**a’**) untextured; (**b**,**b’**) samples with dimple area density of 10%; (**c**,**c’**) samples with dimple area density of 20% and (**d**,**d’**) samples with dimple area density of 40%.

Figure 6a–d present the wear morphologies of untextured and textured steel surfaces with dimple area densities of 10%, 20%, and 40% sliding against POM after wear. Grooves and pits appeared on the untextured worn steel surface. The black areas on the untextured worn steel surface indicate adhesive wear scars that may be caused by carbonation due to high pressure and temperature. The textured steel surfaces obviously had fewer scratches, indicating that the contact conditions improved. However, the wear around the edge of the dimples was much more severe and may be caused by stress concentration. Along the sliding direction in the textured steel specimens after wear, unevenly distributed black areas were observed, which may be due to polymeric material transfer and will be further studied by EDX analysis. Figure 6a’–d’ represent the surface morphologies of the mating POM counterfaces corresponding to Figure 6a–d. As the dimple area density increased, the grooves became wider and deeper, which was believed to contribute to the increase in wear rates (Figure 5a).

**Figure 7:**
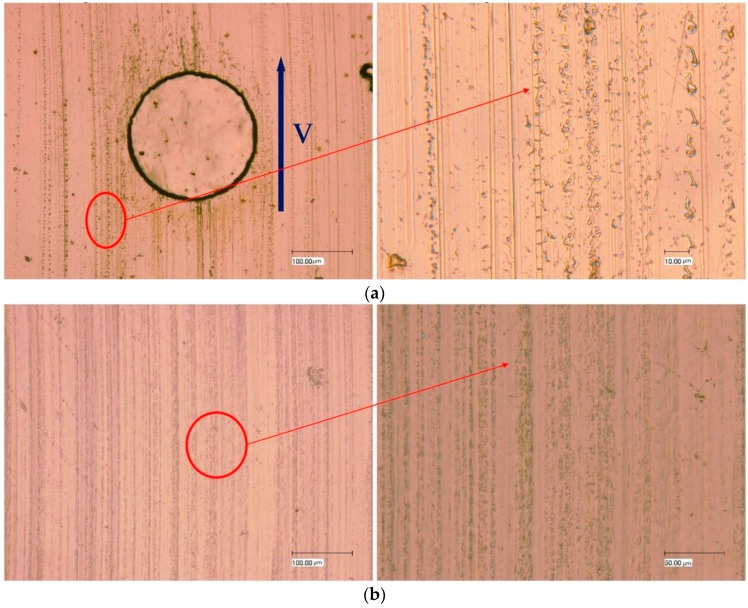
(**a**) The textured steel surface (dimple area density of 10%, dimple depth of 5 μm) and (**b**) the corresponding mating POM surface.

Figure 7 displays the steel surface and the corresponding mating POM surface when the dimple area density is 10% and the dimple depth is 5 μm. At this condition, the wear rate was the lowest among the tribo-pairs of POM/steel, showing a reduced wear effect (Figure 5b) compared to untextured tribo-pairs. It can be seen that small protuberances exist on the steel around the edges of the grooves. These protuberances were possibly the transferred POM, which may benefit from the wear reduction and was further analyzed through EDX. The corresponding mating POM surface (Figure 7b) exhibited fine and shallow grooves. Protuberances were observed around the grooves.

**Figure 8:**
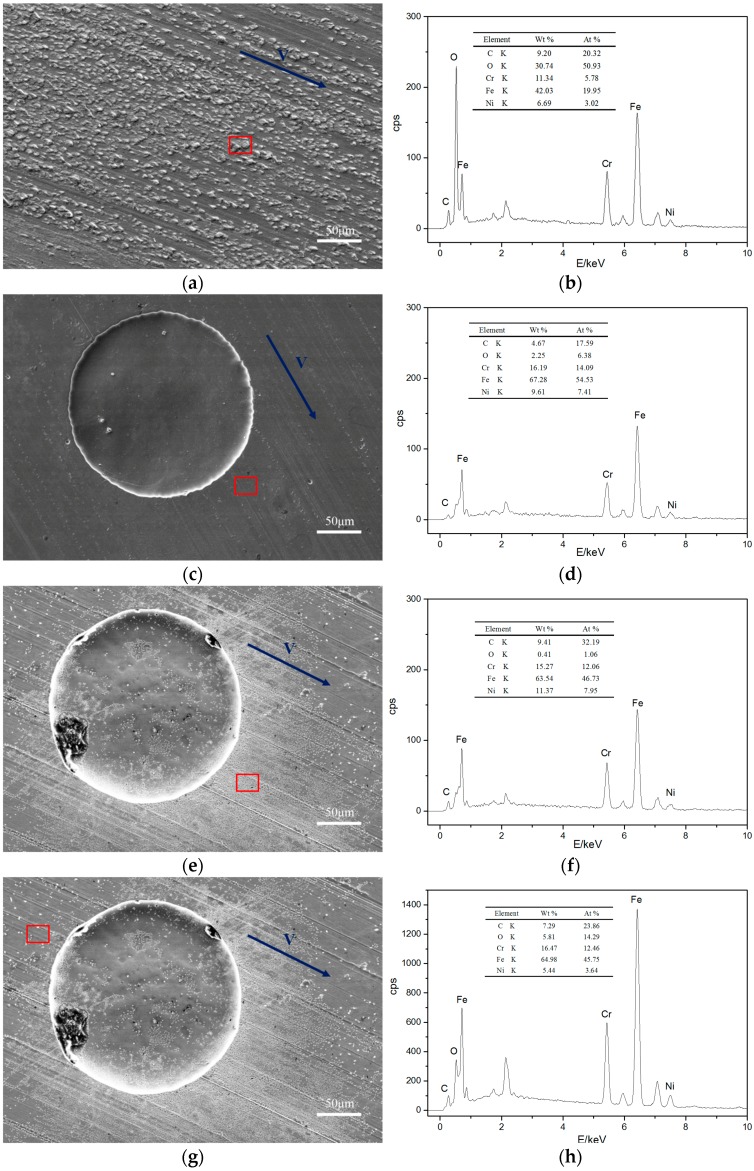
The SEM images of stainless steel surface against POM and corresponding EDX analysis: (**a**) an untextured steel surface and (**b**) the corresponding EDX analysis; (**c**) a textured steel surface (dimple area density of 10%, dimple depth of 5 μm) and (**d**) the corresponding EDX analysis; (**e**) the area on the sample (dimple area density of 10%, dimple depth of 10 μm) and (**f**) the corresponding EDX analysis; (**g**) the area on the textured steel surface (dimple area density of 10%, dimple depth of 10 μm) and (**h**) the corresponding EDX analysis.

Figure 8a,b present SEM images of untextured steel surfaces sliding against POM after wear and a corresponding EDX analysis of the red frame area. The untextured surface exhibited many scaly protuberances. The elements C and O on the untextured surface were higher, suggesting that POM was transferred to its mating steel surface. As shown in the SEM images (Figure 8c) of the steel surface with textures of the dimple area densities of 10% and dimple depths of 5 μm, the worn surface was smooth, and a few scratches and fine humps existed. Compared to the non-textured samples, the contents of the elements of C and O decreased (Figure 8d), denoting that the adhesion and transferring of POM were weakened in the wear process. Meanwhile, on the steel surface (textures with dimple area densities of 10% and dimple depths of 10 μm (Figure 8e)), the areas around the dimples were severely worn, indicating that the tribo-pair had experienced bad wear conditions. The EDX analysis (Figure 8f) showed a higher content of element C and lower element O at the area indicated by the red frame area compared to Figure 8c,d, implying that POM underwent severe burn and material transfer during the wear process. The EDX analysis of the area (Figure 8g) revealed a lower element C and higher element O content compared to those areas in Figure 8e, suggesting a weakened burn and wear of POM.

**Figure 9:**
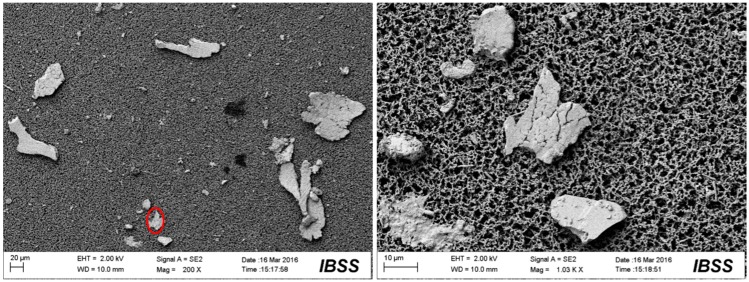
SEM images of the wear debris found in UHMWPE/untextured steel after wear.

**Figure 10:**
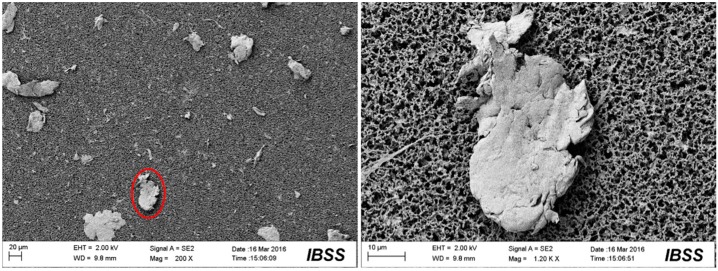
SEM images of the wear debris found in UHMWPE/textured steel (dimple area density of 10%, dimple depth of 10 μm) after wear.

**Figure 11:**
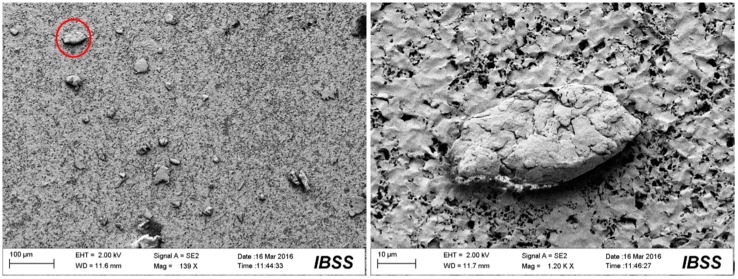
SEM images of the wear debris found in UHMWPE/textured steel (dimple area density of 20%, dimple depth of 10 μm) after wear.

**Figure 12:**
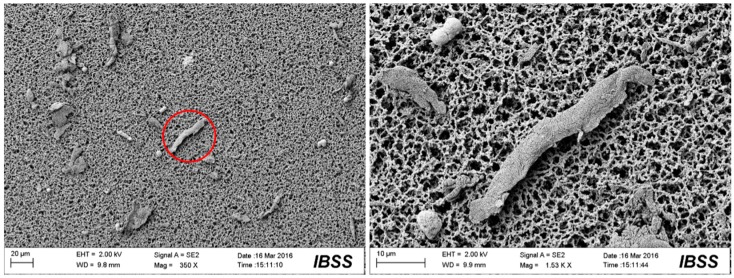
SEM images of the wear debris found in UHMWPE/textured steel (dimple area density of 40%, dimple depth of 10 μm) after wear.

Figure 9, Figure 10, Figure 11 and Figure 12 show the surface morphologies of the wear debris found in the tribo-pairs after wear. The wear debris (Figure 9) found in the UHMWPE/untextured steel had a flake shape and a smooth surface. The wear debris (Figure 10) presented scaly wear scars and was more irregular for UHMWPE/steel with a dimple area density of 10% and dimple depth of 10 μm and the dimples showed effects in reducing wear. The wear debris (Figure 11) revealed a crumby shape and indicated that the material was peeling off for the UHMWPE/steel with a dimple area density of 20%. When the wear rate increased (UHMWPE/steel with dimple area density of 40% and dimple depth of 10 μm), the wear debris (Figure 12) had become rod-shaped.

### 3.4. Simulation

**Figure 13:**
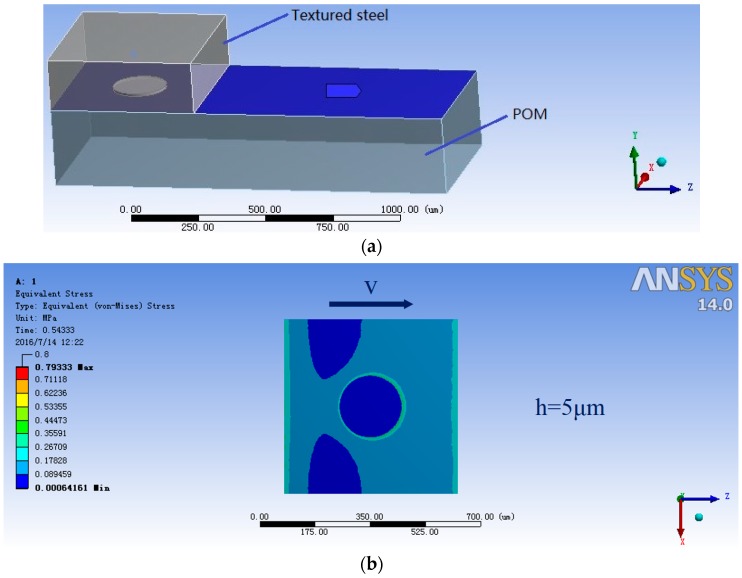

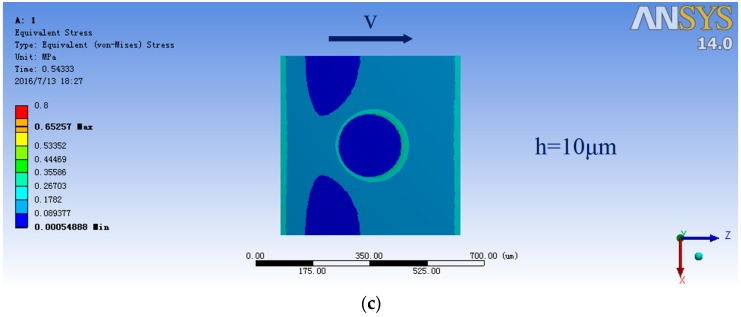
(**a**) Finite element simulation of the mating surfaces of steel against POM; (**b**) a single dimple’s pressure distribution chart of steel with dimple depth of 5 μm and (**c**) 10 μm.

To further investigate wear around dimples edges, a finite element simulation was carried out in ANSYS workbench. The contact of mating between steel and POM was simplified as a dimple block sliding against a plane with solid contact (Figure 13a), where textured steel was assumed to work as the plane and POM as the block. Their surface roughness was ignored. Figure 13b,c shows a single dimple’s pressure distribution chart for steel with a dimple depth of 5 μm and 10 μm, respectively. The areas of the dimples along the movement of mating surfaces exhibited a severe stress distribution, and so it can be speculated that these areas would experience more stress in the articulating process and produce much more cutting wear effects to its counterface. The simulation analysis showed that the affected area, due to the stress concentration of the dimples on the sample with a dimple depth of 5 μm (Figure 13b), was much smaller than that of a sample with dimple depth of 10 μm (Figure 13c), which probably contributed to the lower wear rate of the samples with a dimple depth of 5 μm.

## 4. Discussion

This paper investigated the effects of artificially fabricated surface textures on tribo-pairs of steel sliding against polymer materials. A reduction of the wear rate was observed, which can probably be attributed to the effect of the dimples on trapping wear debris. The coefficient of friction in this study indicates a mixed lubrication mode, which means that one part of the mating surface is solid contact, and hydrodynamic lubrication exerts in another area. The hydrodynamic lubrication effect may help reduce wear. The adhesive behavior and transfer of POM were weakened (Figure 8c,d), thus possibly reducing the wear rates. In this way, less production of wear debris probably resulted in an incomplete polymeric transfer layer, which caused an increase in the friction coefficient.

On the other hand, an increase in the wear rate was also obtained. Increased wear may be due to the stress concentration of dimples revealed by simulated results. The results indicated that for the given tribo-pair, surface textures can affect wear rates and thus wear resistance. The optimized design of the parameters of surface textures will improve the wear properties and prolong the life of the tribological contacts of steel sliding against soft materials.

It can be indicated from Figure 5 that with increasing elastic modulus, wear rates decreased for non-textured tribo-pairs. This is because high elastic modulus indicates high resistance against wear. This is why wear rate of PEEK/textured steel was less than that of UHMWPE/textured steel. Although POM is stiffer than UHMWPE, the wear rate of POM/textured steel was greater than that of UHMWPE/textured steel when dimple depth was 10 μm. The above results implied that elastic modulus of the counterpart as well as artificial manufactured surface textures affected wear performance of the tribo-pairs studied.

**Figure 14:**
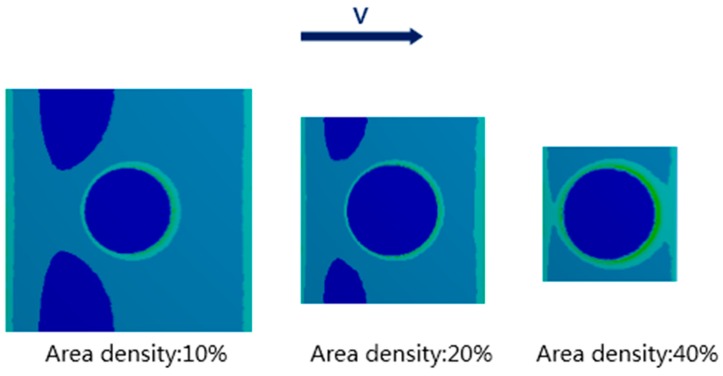
Finite element simulation of the mating surfaces of steel against POM at dimple area density of 10%, 20% and 40%.

Dimples area density and depth are two important parameters influencing tribological properties of textured tribo-pairs [2]. Take POM/textured steel for example, when dimple depth was 10 μm, with increasing dimples area density, the affected area of stress concentration increased as well (Figure 14), which probably led to the rising wear rate of POM/textured steel (Figure 5). While dimple depth was 5 μm, stress concentration was less than the condition when dimple depth was 10 μm (Figure 13), which may be why the wear rate for POM/textured steel was smaller at dimple depth of 5 μm. Compared to POM/textured steel, UHMWPE/textured steel showed the opposite trend. Its wear rate was higher at dimple depth of 5 μm than that at dimple depth of 10 μm, which was probably due to the difference of material properties between POM and UHMWPE. Correspondingly, the lowest wear rate for POM tribo-pair was at dimple depth of 5 μm and dimples area density of 10%, while that for UHMWPE tribo-pair was at dimple depth of 10 μm and dimples area density of 20%.

Surface morphology analysis has been a commonly used method to inspect tribological contact conditions. The adhesive wear and burn of polymer materials occurred in the articulating process indicated by the surface morphologies (Figure 6a). Surface morphologies after wear had few scratches compared to untextured specimens (Figure 8a), which corresponds to the reduced wear rates of specimens with dimple area densities of 10% and 20% compared to untextured ones (Figure 5b). On the other hand, much severe wear around the edges of the dimples (Figure 6b,c) was observed, which likely was caused by stress concentration.

FEA (Figure 13 and Figure 14) revealed that surface textures possibly caused the stress concentration around the edges of the dimples and thus resulted in stronger forces on the soft counterfaces. For the non-textured tribo-pairs, the steel surface was covered by a large area of polymer transfer film (Figure 8a,b), while the wear debris presented flake-like morphologies (Figure 9), which may be peeled off from the complete transfer film. As the number of dimples increased corresponding to the dimple area density, the affected areas increased as revealed by FEA (Figure 14). The strengthened affected areas worked like blades between the articulating surfaces. When there were more blades, the cutting effects were stronger, and thus the wear debris tended to reveal micro-cutting morphologies (Figure 12). For the wear-reduced condition, the wear debris were smaller and lumpy. When wear increased, the wear debris were rod-shaped or twisted due to the stronger cutting effects involved. The wear debris had different surface morphologies at varying tribo-pairs, indicating different wear mechanisms.

## 5. Conclusions

This study has investigated the wear behavior of friction pairs consisted of soft materials, which are less studied in current literature. A thorough understanding of wear behavior of the soft counterfaces is achieved. Based on the analyzed techniques in the study, it can be concluded that wear behavior relates to the elastic modulus of soft materials and parameters of surface textures. Polymeric material transfer occurred, which may participate in adhesive wear. The wear debris found in the tribo-pairs with varying surface textures present different surface morphologies. Non-textured tribo-pair revealed flaky and smooth wear debris. With reduced wear, the wear debris tended to be lumpy while at increased wear, the wear debris tended to be rod-shaped or twisted.

## Figures and Tables

**Table 1:** The materials’ properties.

Materials	Density (g·cm^−3^)	Elastic Modulus (GPa)	Poissons’ Ratio
304 stainless steel	7.93	193	0.26
UHMWPE	0.95	1.1	0.41
POM	1.40	2.6	0.39
PEEK	1.36	3.66	0.4

UHMWPE: ultrahigh molecular weight polyethylene; POM: polyoxymethylene; PEEK: polyetheretherketone.

**Table 2:** The geometrical parameters of the dimples.

Dimples Area Density	Dimples Depth	Dimples Diameter
10%	5 μm	200 μm
20%	10 μm	-
40%	-	-

## References

[1] Gropper D., Wang L., Harvey T.J. (2016). Hydrodynamic lubrication of textured surfaces: A review of modeling techniques and key findings. Tribol. Int..

[2] Ibatan T., Uddin M.S., Chowdhury M.A.K. (2015). Recent development on surface texturing in enhancing tribological performance of bearing sliders. Surf. Coat. Technol..

[3] Brizmer V., Kligerman Y., Etsion I. (2003). A laser surface textured parallel thrust bearing. Tribol. Trans..

[4] Etsion I., Halperin G., Brizmer V., Kligerman Y. (2004). Experimental investigation of laser surface textured parallel thrust bearings. Tribol. Lett..

[5] Dadouche A., Conlon M.J. (2016). Operational performance of textured journal bearings lubricated with a contaminated fluid. Tribol. Int..

[6] Tala-Ighil N., Fillon M. (2015). A numerical investigation of both thermal and texturing surface effects on the journal bearings static characteristics. Tribol. Int..

[7] Grabon W., Koszela W., Pawlus P., Ochwat S. (2013). Improving tribological behaviour of piston ring–cylinder liner frictional pair by liner surface texturing. Tribol. Int..

[8] Ryk G., Etsion I. (2006). Testing piston rings with partial laser surface texturing for friction reduction. Wear.

[9] Wang X., Kato K., Adachi K., Aizawa K. (2003). Loads carrying capacity map for the surface texture design of SiC thrust bearing sliding in water. Tribol. Int..

[10] Wang X., Kato K. (2003). Improving the Anti-seizure Ability of SiC Seal in Water with RIE Texturing. Tribol. Lett..

[11] Etsion I., Halperin G. (2002). A laser surface textured hydrostatic mechanical seal. Tribol. Trans..

[12] Etsion I., Kligerman Y., Halperin G. (1999). Analytical and experimental investigation of laser-textured mechanical seal faces. Tribol. Trans..

[13] Hu J., Xu H. (2016). Friction and wear behavior analysis of the stainless steel surface fabricated by laser texturing underwater. Tribol. Int..

[14] Braun D., Greiner C., Schneider J., Gumbsch P. (2014). Efficiency of laser surface texturing in the reduction of friction under mixed lubrication. Tribol. Int..

[15] Tang W., Zhou Y., Zhu H., Yang H. (2013). The effect of surface texturing on reducing the friction and wear of steel under lubricated sliding contact. Appl. Surf. Sci..

[16] He B., Chen W., Wang Q.J. (2008). Surface texture effect on friction of a microtextured poly (dimethylsiloxane)(PDMS). Tribol. Lett..

[17] Li J., Zhou F., Wang X. (2011). Modify the friction between steel ball and PDMS disk under water lubrication by surface texturing. Meccanica.

[18] Li M., Huang W., Wang X. (2015). Bioinspired, peg-studded hexagonal patterns for wetting and friction. Biointerphases.

[19] Korpela T., Suvanto M., Pakkanen T.T. (2015). Wear and friction behavior of polyacetal surfaces with micro-structure controlled surface pressure. Wear.

[20] Xiong D., Qin Y., Li J., Wan Y., Tyaji R. (2015). Tribological properties of PTFE/laser surface textured stainless steel under starved oil lubrication. Tribol. Int..

[21] Zhang B., Huang W., Wang X. (2012). Biomimetic surface design for ultrahigh molecular weight polyethylene to improve the tribological properties. Proc. Inst. Mech. Eng. Part J J. Eng. Tribol..

[22] Zhang B., Huang W., Wang J., Wang X. (2013). Comparison of the effects of surface texture on the surfaces of steel and UHMWPE. Tribol. Int..

